# Ocular growth and metabolomics are dependent upon the spectral content of ambient white light

**DOI:** 10.1038/s41598-021-87201-2

**Published:** 2021-04-07

**Authors:** Raymond P. Najjar, Juan Manuel Chao De La Barca, Veluchamy A. Barathi, Candice Ee Hua Ho, Jing Zhan Lock, Arumugam R. Muralidharan, Royston K. Y. Tan, Chetna Dhand, Rajamani Lakshminarayanan, Pascal Reynier, Dan Milea

**Affiliations:** 1 Singapore Eye Research Institute, Singapore, Singapore; 2 The Ophthalmology and Visual Sciences ACP, Duke-NUS Medical School, Singapore, Singapore; 3 Département de Biochimie et Génétique, Centre Hospitalier Universitaire d’Angers, Angers, France; 4 Unité Mixte de Recherche MITOVASC, CNRS 6015, INSERM U1083, Université d’Angers, Angers, France; 5 Department of Ophthalmology, Yong Loo Lin School of Medicine, National University of Singapore, Singapore, Singapore; 6 Department of Ocular Bio-Engineering, National University of Singapore, Singapore, Singapore; 7 CSIR-Advanced Materials and Processes Research Institute, Hoshangabad Road, Bhopal, 462026 India; 8 Singapore National Eye Center, Singapore, Singapore

**Keywords:** Visual system, Refractive errors, Metabolomics

## Abstract

Myopia results from an excessive axial growth of the eye, causing abnormal projection of remote images in front of the retina. Without adequate interventions, myopia is forecasted to affect 50% of the world population by 2050. Exposure to outdoor light plays a critical role in preventing myopia in children, possibly through the brightness and blue-shifted spectral composition of sunlight, which lacks in artificial indoor lighting. Here, we evaluated the impact of moderate levels of ambient standard white (SW: 233.1 lux, 3900 K) and blue-enriched white (BEW: 223.8 lux, 9700 K) lights on ocular growth and metabolomics in a chicken-model of form-deprivation myopia. Compared to SW light, BEW light decreased aberrant ocular axial elongation and accelerated recovery from form-deprivation. Furthermore, the metabolomic profiles in the vitreous and retinas of recovering form-deprived eyes were distinct from control eyes and were dependent on the spectral content of ambient light. For instance, exposure to BEW light was associated with deep lipid remodeling and metabolic changes related to energy production, cell proliferation, collagen turnover and nitric oxide metabolism. This study provides new insight on light-dependent modulations in ocular growth and metabolomics. If replicable in humans, our findings open new potential avenues for spectrally-tailored light-therapy strategies for myopia.

## Introduction

Emmetropization is the process that controls ocular growth rate and ensures that the size of the eye is harmonized with its focal power^1^. Aberrant axial ocular growth is associated with refractive error development. While reduced axial elongation leads to hyperopia (long-sightedness), increased axial length leads to myopia (short-sightedness). Myopia is the leading cause of vision impairment worldwide^2^. It affects 20–39% of Caucasians^3,4^ and its prevalence can rise up to alarming magnitudes (> 80%) in East and Southeast Asian countries like Taiwan, Hong Kong and Singapore^5–11^. Besides its direct and indirect socio-economic burdens^12,13^, high myopia (spherical equivalent of − 6.00 Diopters or worse), affecting 2.7% of the world population^14^, is associated with increased risk of ocular complications leading to irreversible vision loss such as glaucoma, retinal detachment, macular degeneration and choroidal neovascularization^15,16^.

Amongst various genetic and environmental triggers influencing ocular growth and myopia onset^17,18^, reduced time spent outdoors has recently been singled-out as a major risk factor^19–21^. The protective effect of time spent outdoors is independent of physical activity and could be due to the intensity and unique spectral characteristics of sunlight, which lack in artificial indoor lighting^22^. While increased time spent outdoors is efficient in preventing the onset of the disease in humans^23,24^, studies show that exposure to bright light can limit or halt the development of myopia in animal models^25–27^. Unfortunately, increasing time outdoors for children remains difficult, particularly during school time, given weather conditions and the cultural commitment to educational success in many parts of the world. For instance, children of Chinese ethnicity living in Singapore still spend 10.7 h less time outdoors per week and are 26% more at risk of becoming myopic than children of Chinese ethnicity living in Sydney^20^.

The impact of the spectral composition of light on emmetropization and homeostatic control of ocular growth in humans remains unclear, however, evidence for a role of color vision in the control of eye growth has been reported in a school-based study showing that the incidence of myopia is lower in children with protan or deutan color deficiencies compared to children with normal color vision^28^. Similarly, lab-based studies highlighted a decreased sensitivity to low spatial frequency short-wavelength cones (S-cone) stimuli in myopes compared to emmetropes^29^, and a correlation between refraction and the relative sensitivity of the accommodation system to long-wavelength cones (L-cones) contrast^30^. In animal models, findings from our lab and others suggest that modulating the spectral composition of light can affect ocular growth and refractive error development. For instance, in fish, squid, guinea pigs and chickens, eyes become more myopic under long wavelength light and more hyperopic under short wavelength light^22,31–36^, and exposing chickens to narrow-band blue light reduces the development of form-deprivation myopia^34^. Furthermore, work from Rucker et al. highlighted an important role of the short-wavelength content of white light in the control of eye growth, myopia development and choroid thickness under low temporal frequency lighting conditions^37,38^. On the other hand, reports from rhesus macaques and tree shrews suggest that exposure to red light promotes hyperopia^39–41^, and Liu and colleagues found no significant differences in refraction in rhesus macaques reared under blue light (455 nm) compared to white light^42^. While there is some consensus over major roles of dopamine and potentially nitric oxide (NO) in the protective effect of bright light on myopia^25,43–45^, the molecular and metabolic pathways involved in the spectral selectivity of ocular growth remain unclear.

In this study, we investigated the impact of moderate levels of blue-enriched white (BEW) light on ocular growth and metabolomics profiles in a chicken model of form-deprivation myopia. We report that, compared to standard white light (SW), BEW light can slow the development and accelerate recovery from aberrant axial elongation induced by form-deprivation. In addition, the spectral composition of equiluminant lights can modulate the metabolomic profile within the retina and vitreous of form-deprived (FDEP) and control eyes.

## Results

**Figure 1:**
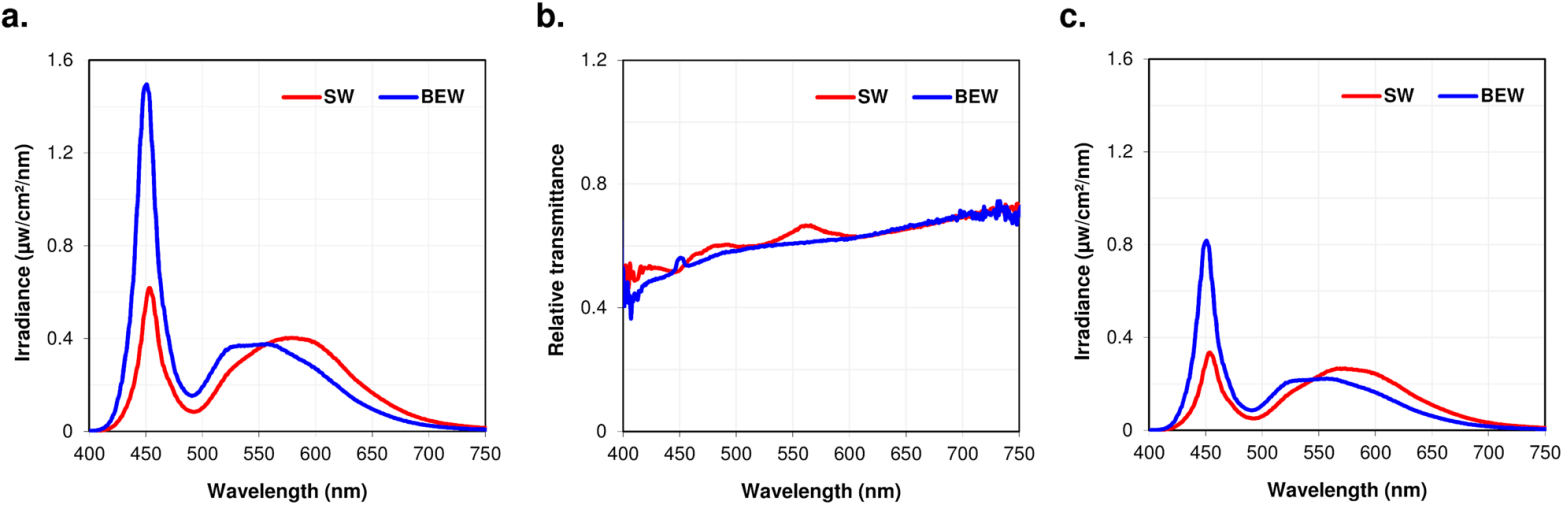
Spectral composition of light reaching control and FDEP eyes. (**a**) Spectral composition of the lighting environments in both SW and BEW groups measured at the corneal level of control eyes. (**b**) Relative spectral transmittance of the diffusers used for form-deprivation. (**c**) Spectral composition of the lighting environments in both SW and BEW groups measured with the diffuser at the corneal level of a FDEP eye.

**Figure 2:**
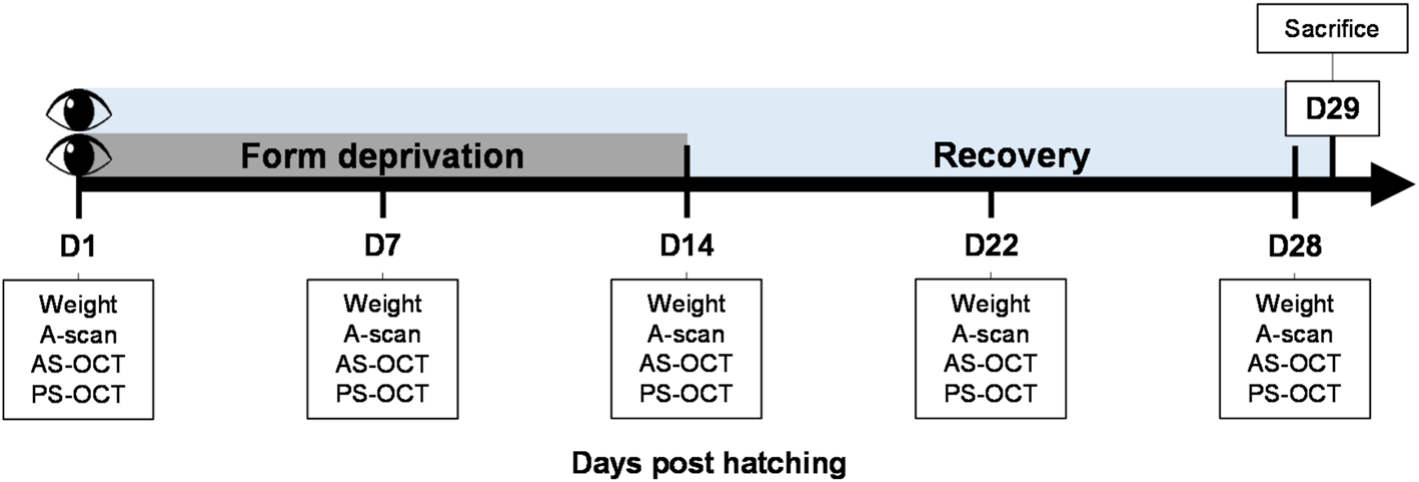
Experimental protocol. Thirty-six, 1-day old chicks were randomly assigned to two groups of 18 animals each and raised under SW or BEW light emitting diode (LED) lighting for 29 days. Animals were randomly monocularly fitted with a diffuser on D1. Diffusers were removed on D14 and FDEP eyes recovered until D29. Ophthalmic examinations were performed on D1, D7, D14, D22 and D28. On D29, animals were sacrificed and eyes were enucleated. The retinas and vitreous were harvested for metabolomic analysis. Ten eyes were dissected for histological evaluation of the retina, choroid and sclera. *AS-OCT* anterior segment optical coherence tomography, *PS-OCT* posterior segment optical coherence tomography.

Thirty-six chicks (Lohmann Brown) were randomly separated into four batches of 9 animals each. Batches 2 and 4 (group 1, n = 18, 9 males) were reared under SW (3900 K, average ± standard deviation (SD): 233.1 ± 18.2 lux) light while batches 1 and 3 (group 2, n = 18, 9 males) were reared under BEW (9700 K, 223.8 ± 19.6 lux) light (see Fig. 1 and Table 1 for more details on lighting characteristics). Form-deprivation was induced monocularly in all chicks using a frosted diffuser from day 1 post-hatching (D1, baseline) until D14. Recovery from form-deprivation was evaluated between D14 and D28. Ocular axial length (AL), choroidal and retinal thicknesses, as well as anterior segment parameters were measured using ultrasonography and optical coherence tomography (OCT) on D1, D7, D14, D22, and D28, and compared between groups and eyes. Histological thickness measurements of the choroid, retina and sclera, as well as targeted metabolomic analyses of vitreous and retinas were performed after sacrifice on D29 (Fig. 2).

**Table 1:** Characteristics of experimental lighting environments in both groups.

	Standard white	Blue-enriched white
**Without diffuser**		
Corneal illuminance (lux)	233.1	223.8
Irradiance (µW/cm^2^)	70.88	85.77
Photon flux (1/cm^2^/s)	1.99E + 14	2.23E + 14
Log photon flux (log_10_ (1/cm^2^/s)	14.30	14.35
**With diffuser**		
Corneal illuminance (lux)	148.1	135.7
Irradiance (µW/cm^2^)	43.81	49.68
Photon flux (1/cm^2^/s)	1.24E + 14	1.31E + 14
Log photon flux (log_10_ (1/cm^2^/s)	14.09	14.12

### Weights

Overall growth and weights were not different between groups across the 28-days experimental period (*P* > 0.05) (Supplementary Fig. S1).

### Aberrant axial elongation induced by form-deprivation is attenuated by BEW light

**Figure 3:**
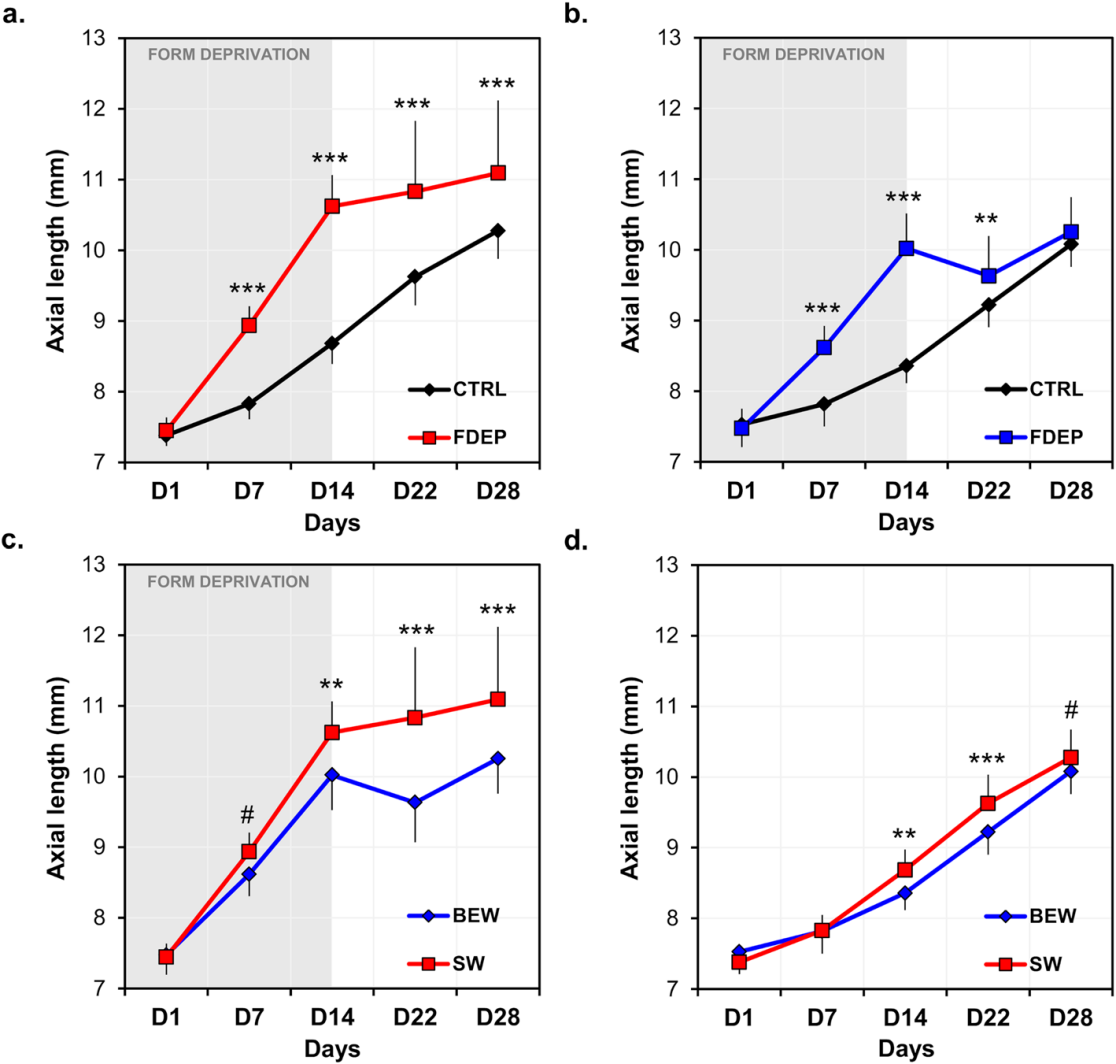
Axial length of FDEP and control eyes in animals reared under BEW (n = 18) or SW (n = 18) lights. After 14 days of form-deprivation, AL was increased in FDEP eyes compared to control eyes in the SW (**a**) and BEW (**b**) groups (P < 0.001). Two weeks following form-deprivation (D28), the AL of FDEP eyes was no longer different from control eyes in chicks reared under BEW light but not in animals reared under SW light (**a**,**b**). FDEP eyes of animals raised under BEW light displayed shorter AL compared FDEP eyes of animals raised under SW light (P < 0.001) (**c**). BEW attenuated axial elongation due to form-deprivation observed on D14 (*P* = 0.002) (**c**). Control eyes reared under BEW light displayed shorter AL than eyes reared under SW light on D14 and D22 (*P* = 0.002 and *P* < 0.001, respectively) (**d**). Data are represented as average ± SD. Post hoc pairwise comparison significance: ^#^*P* < 0.1; *P < 0.05; **P < 0.01; ***P < 0.001.

AL was significantly increased in the FDEP eyes compared to control eyes in both groups [SW: (*F*(1, 34) = 57.45, *P* < 0.001); BEW: (*F*(1, 34) = 35.32, *P* < 0.001)] (Fig. 3a,b, Table 2). This increase in AL was dependent upon the upon the days of the experiment (SW: *F*(4, 34) = 28.00, *P* < 0.001; BEW: *F*(4, 34) = 60.86, *P* < 0.001). One week subsequent to form-deprivation (D22), AL of FDEP eyes exposed to BEW light was reduced by 0.39 ± 0.36 mm compared to D14 and was only 0.41 ± 0.43 mm longer than the control eyes (*P* = 0.002) (Fig. 3b, Table 2). This reduction in AL was not observed in animals reared under SW light who displayed an increase of 0.21 ± 0.7 mm on D22 compared to D14. By D28, average AL of FDEP eyes remained different from control eyes in animals reared under SW light (*P* < 0.001; Fig. 3a) but not in animals reared under BEW light (*P* = 0.18; Fig. 3b, Table 2).

**Table 2:** In vivo ocular measurements in control and form-deprived eyes exposed to standard white (SW) or blue enriched white (BEW) light.

Ocular parameters	Condition	Days					*P*-values 2wRM-ANOVA		
		D1	D7	D14	D22	D28	Group	Day	Group × Day
**Control eyes**									
Axial length (mm)	SW	7.38 ± 0.15	7.83 ± 0.22	8.68 ± 0.29	9.63 ± 0.41	10.3 ± 0.40	0.07	< 0.001	< 0.001
	BEW	7.53 ± 0.32	7.82 ± 0.32	8.36 ± 0.24**	9.22 ± 0.32***	10.1 ± 0.32^#^			
Choroidal thickness (µm)	SW	189.3 ± 60.3	173.5 ± 50.0	191.5 ± 32.7	232.3 ± 39.0	217.9 ± 37.9	0.03	< 0.001	0.04
	BEW	183.5 ± 54.8	227.8 ± 37.0**	222.7 ± 39.3	222.3 ± 23.7	249.2 ± 26.5*			
Retinal thickness (µm)	SW	287.7 ± 11.8	285.9 ± 21.5	283.4 ± 12.0	279.1 ± 13.1	263.1 ± 17.8	0.18	< 0.001	0.24
	BEW	285.9 ± 8.50	298.5 ± 14.4	285.1 ± 17.5	277 ± 9.60	274.6 ± 16.3			
ACD (µm)	SW	907.8 ± 44.7	1124.3 ± 79.1	1222.8 ± 55.0	1406.7 ± 63.2	1507.2 ± 65.4	0.46	< 0.001	0.04
	BEW	894.9 ± 45.2	1112.2 ± 45.7	1251.1 ± 50.4	1380.6 ± 50.8	1478.1 ± 61.6			
CCT (µm)	SW	166.7 ± 8.60	182.3 ± 9.60	194.8 ± 11.7	207.1 ± 13.6	214.2 ± 19.2	0.53	< 0.001	0.25
	BEW	172.0 ± 10.9	179.2 ± 10.5	190.1 ± 10.4	205.3 ± 14.4	209.4 ± 12.4			
**Form-deprived eyes**									
Axial length (mm)	SW	7.45 ± 0.19	8.94 ± 0.27	10.6 ± 0.44	10.8 ± 1.00	11.1 ± 1.03	< 0.001	< 0.001	< 0.001
	BEW	7.47 ± 0.28	8.62 ± 0.31^#^	10.0 ± 0.50**	9.63 ± 0.56***	10.3 ± 0.49***			
Choroidal thickness (µm)	SW	185.5 ± 57.1	155.7 ± 40.0	175.0 ± 35.5	451.7 ± 180.2	464.7 ± 158.7	0.22	< 0.001	0.80
	BEW	183.5 ± 54.8	190.2 ± 59.3	186.0 ± 46.3	524.6 ± 43.2	501.1 ± 85.2			
Retinal thickness (µm)	SW	284 ± 14.1	259.3 ± 23.4	263.2 ± 13.0	271.4 ± 16.5	270.1 ± 22.6	0.59	< 0.001	0.001
	BEW	285.9 ± 8.5	282.1 ± 12.2*	245.1 ± 29.4*	287.2 ± 13.2	274.8 ± 11.0			
ACD (µm)	SW	898.7 ± 60.1	1286.7 ± 169.1	1811.1 ± 302.3	1861.1 ± 219.5	1930.6 ± 243.4	0.008	< 0.001	< 0.001
	BEW	914.8 ± 100.6	1138.7 ± 134.1*	1580.0 ± 270.1**	1669.4 ± 244.4**	1672.5 ± 231.1***			
CCT (µm)	SW	165.4 ± 9.70	178.9 ± 12.5	174.3 ± 11.5	180.9 ± 12.0	191.6 ± 10.3	0.03	< 0.001	0.06
	BEW	170.2 ± 10.4	179.0 ± 10.4	180.6 ± 13.7	194.8 ± 18.6	201.5 ± 17.2			

Data are presented as average ± SD. *P*-values represent the significance of the two way repeated measures ANOVA (2wRM-ANOVA) for “group”, “day” and “group × day” comparisons. Post hoc comparisons using Holm Sidak method are shown in this table for “group x day” interactions where ^#^*P* < 0.1; *P < 0.05; **P < 0.01; ***P < 0.001.

Overall, FDEP eyes reared under BEW light had shorter AL compared to FDEP eyes reared under SW light (*F*(1, 34) = 16.43, *P* < 0.001). This difference in AL between groups was dependent upon the days of the experiment (*F*(4, 34) = 11.05, *P* < 0.001). For instance, AL was significantly reduced by 0.60, 1.17 and 0.84 mm on average in FDEP eyes of animals reared under BEW compared to SW light by the end of form-deprivation (D14) (*P* = 0.002), D22 and D28 (*P* < 0.001), respectively (Fig. 3c, Table 2). The AL of control eyes reared under BEW was shorter than control eyes reared under SW light on D14 (*P* = 0.002) and D22 (*P* < 0.001) of the experiment (Interaction Group × Day: *F*(4, 34) = 13.77, *P* < 0.001) (Fig. 3d, Table 2).

### Choroidal and retinal thicknesses in vivo

**Figure 4:**
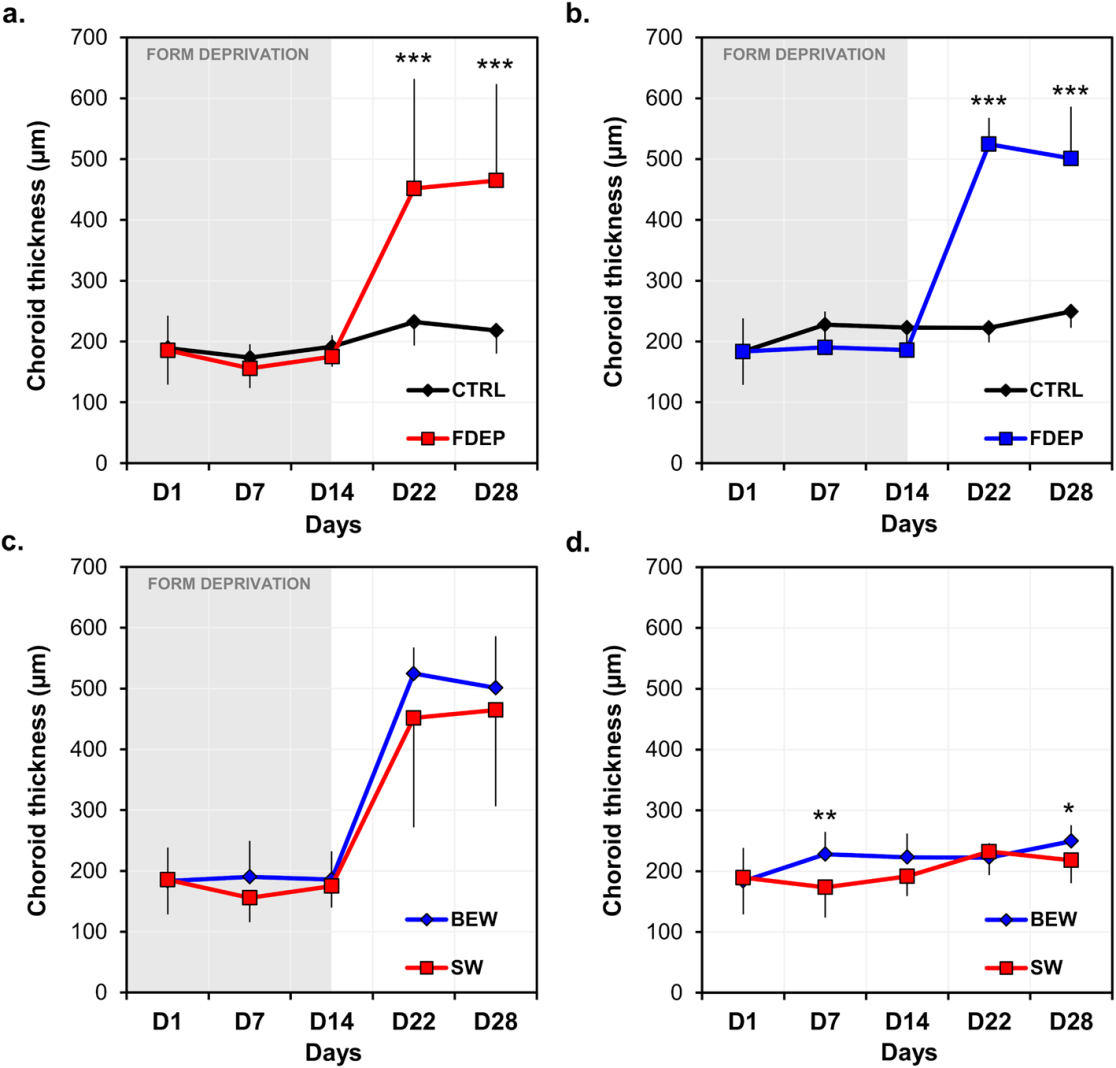
Choroidal thickness of FDEP and control eyes in animals reared under BEW (n = 18) or SW (n = 18) lights. During recovery from form-deprivation, choroidal thickness was increased in FDEP eyes compared to control eyes in the SW (**a**) and BEW (**b**) groups (*P* < 0.001). FDEP eyes of animals reared under BEW light showed similar choroidal thicknesses across the experiment compared to FDEP eyes of animals reared under SW light (*P* > 0.05) (**c**). Control eyes reared under BEW light displayed thicker choroids on D7 (*P* = 0.006) and D28 (*P* = 0.02) compared to eyes reared under SW light (**d**). Data are represented as average ± SD. Post hoc pairwise comparison significance: *P < 0.05; **P < 0.01; ***P < 0.001.

Baseline choroidal and retinal thicknesses were only assessed in 8 chickens in the SW group. Given that no experimental procedures were performed on D1 and that animals are from the same strain and breeders, thicknesses were considered similar between groups. When compared on the last day of form-deprivation (D14), choroids of FDEP eyes were significantly thinner compared to control eyes exposed to SW (− 8.0% ± 14.7%; paired *t* test: *P* = 0.03) or BEW (− 15.5% ± 20.5%; paired *t* test: *P* = 0.03). Subsequently to form-deprivation (D22 and D28), choroidal thickness was significantly increased under both lighting conditions in FDEP eyes (SW: *F*(1, 34) = 18.44, *P* < 0.001; BEW: *F*(1, 34) = 34.6, *P* < 0.001 ); Fig. 4a,b, Table 2).

Even though the choroids of FDEP eyes in animals reared under BEW light tended to be thicker than those reared under SW light, on D7 and following form-deprivation (D22 and D28), these differences did not reach statistical significance (Fig. 4c, Table 2). Conversely, choroids of control eyes reared under BEW light were significantly thicker than eyes reared under SW light (*F*(1, 34) = 4.66, *P* = 0.03), especially on D7 (*P* = 0.006) and D28 (*P* = 0.02) (Interaction Group × Day: *F*(4, 34) = 2.70, *P* = 0.04) (Fig. 4d, Table 2). Retinal thicknesses were reduced by form-deprivation under both lighting conditions (Supplementary Fig. S2, Table 2). Differences between groups were dependent on the day of experiment (Supplementary Fig. S2, Table 2).

### Choroidal, retinal and scleral thicknesses ex vivo

Histological measurements performed in 10 animals (SW: n = 6; BEW: n = 4) on D29 showed an increase in the choroidal thickness of recovering FDEP eyes compared to control eyes under both light conditions (Supplementary Fig. S3). This increase in choroidal thickness only reached significance in eyes exposed to BEW light (*P* = 0.008, paired *t* test) (Supplementary Fig. S3). Eyes exposed to BEW light had thicker choroids (Control: 235.3 ± 77.8 μm; FDEP: 617.6 ± 167.1 μm) compared to eyes exposed to SW light (Control: 145.9 ± 44.3 μm; FDEP: 331.9 ± 200.9 μm) (*P* < 0.05, Student’s *t* test, Supplementary Fig. S3). Retinal, cartilaginous and fibrous scleral thicknesses were not different between eyes or groups (Supplementary Fig. S4).

### Structural parameters of the anterior segment in vivo

**Figure 5:**
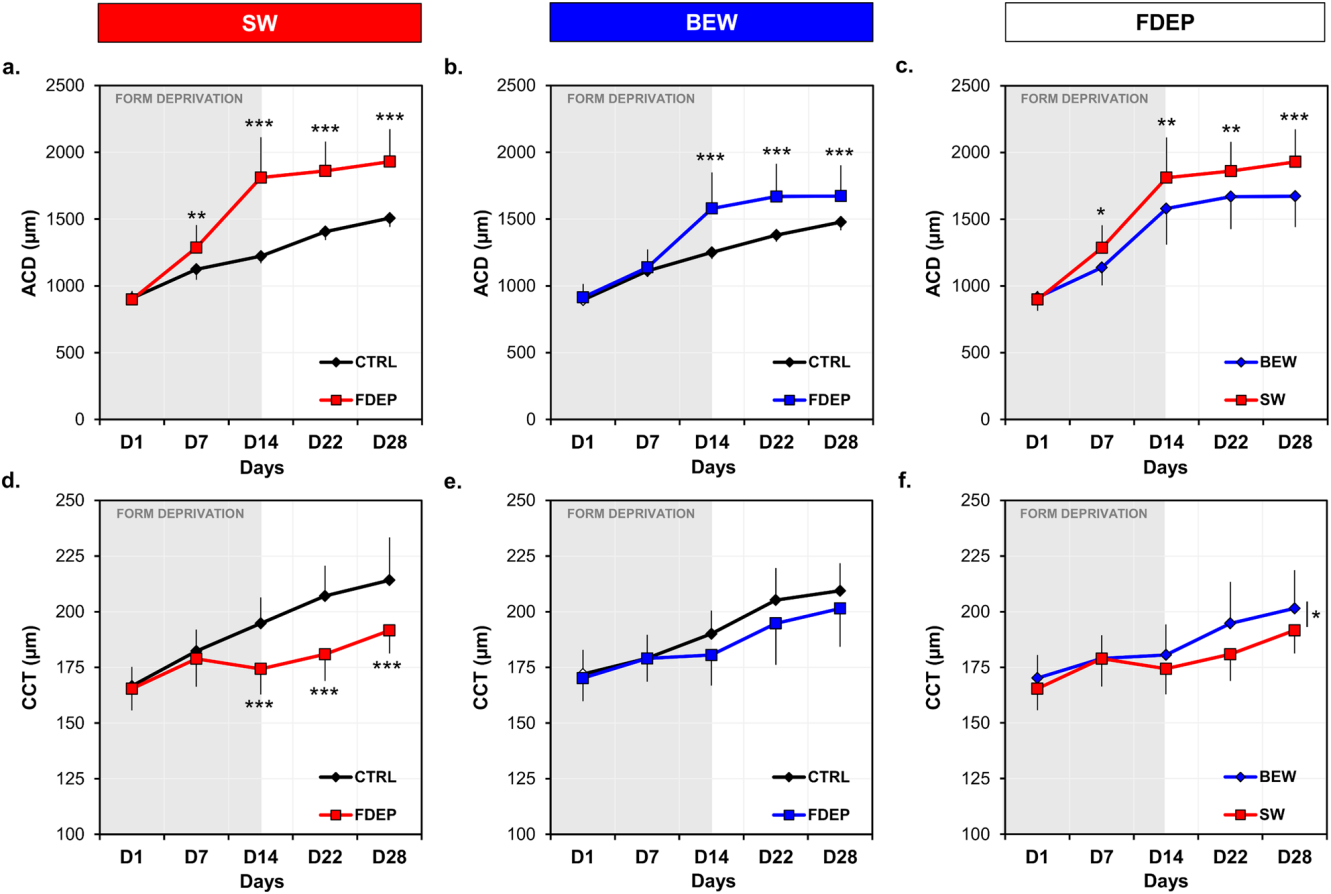
Anterior chamber depth and central corneal thicknesses in FDEP and control eyes under SW and BEW lights. ACD of FDEP eyes were increased compared to control eyes under both lighting conditions (**a**,**b**); however, FDEP eyes reared under BEW showed a reduced ACD compared to FDEP eyes reared under SW light (**c**). CCT of FDEP eyes were reduced under SW light (**d**) but not under BEW light (**e**), compared to control eyes. The CCT of FDEP eyes was reduced under SW light compared to BEW light (*F*(1, 34) = 5.23, *P* = 0.03) (**f**). Data are represented as average ± SD. Post hoc pairwise comparison: *P < 0.05; **P < 0.01; ***P < 0.001.

Anterior chamber depth (ACD) was significantly increased in FDEP eyes, starting D7 under SW light and D14 under BEW light, compared to control eyes [SW: (*F*(4, 34) = 44.10, *P* < 0.001); BEW: (*F*(4, 34) = 21.44, *P* < 0.001)] (Fig. 5a,b, Table 2). This increase in the ACD of FDEP eyes was, however, limited under BEW compared to SW light (*F*(1, 34) = 7.85, *P* = 0.008) (Fig. 5c, Table 2).

Concomitantly, form-deprivation induced a thinning of the cornea on D14, D22 and D28 in animals raised under SW light (*F*(4, 34) = 11.50, *P* < 0.001) but not in those raised under BEW light (Fig. 5d,e, Table 2). Central corneal thickness (CCT) of FDEP eyes was reduced under SW compared to BEW light (*F*(1, 34) = 5.23, *P* = 0.03) (Fig. 5f, Table 2). ACD and CCT of control eyes were not different between groups.

### The vitreal metabolomic profiles of recovering FDEP eyes are different from control eyes and dependent upon the spectral content of the light exposure

**Figure 6:**
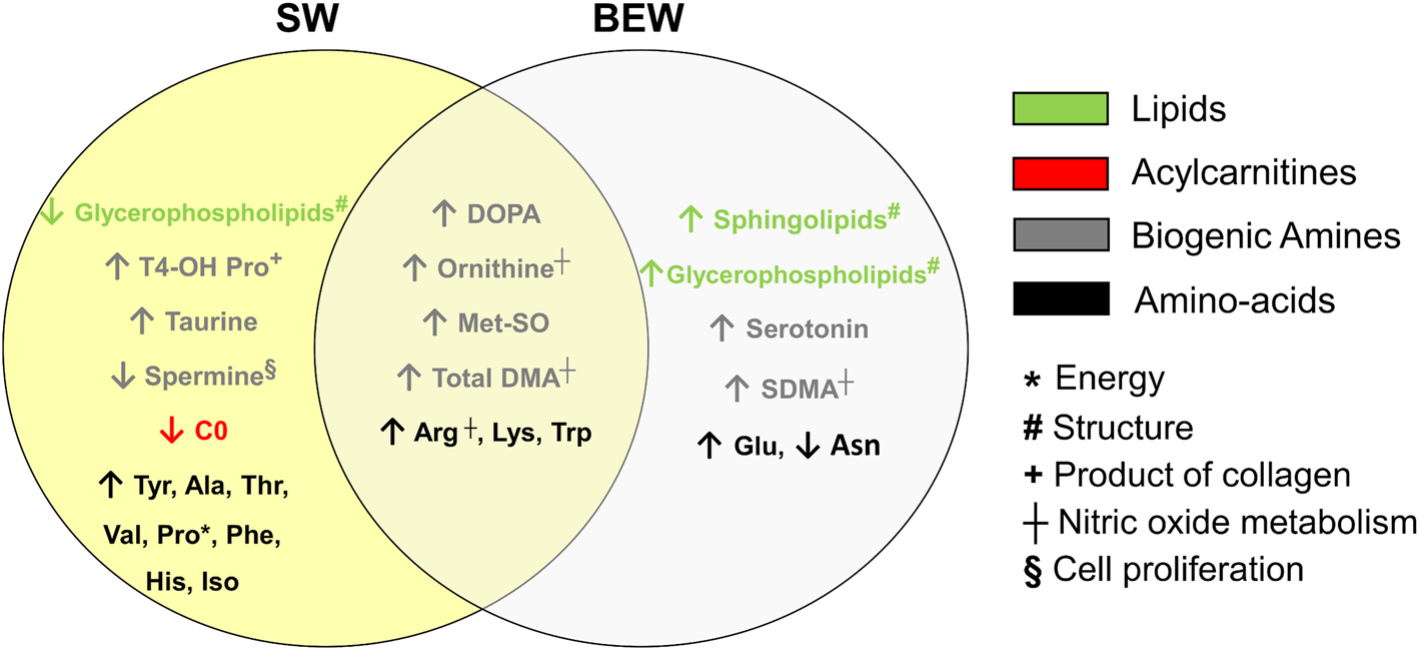
Venn diagram summarizing metabolomic changes in the vitreous of recovering FDEP eyes exposed to SW or BEW light in comparison with control eyes. Significant changes in metabolite levels in FDEP eyes compared to control eyes included increases in biogenic amines and amino acids under both lighting conditions, while glycerophospholipid levels were increased under BEW light and reduced under SW light. *Ala* alanine, *Arg* arginine, *Asn* asparagine, *C0* carnitine, *DOPA* dihydroxyphenylalanine, *Glu* glutamate, *His* histidine, *Iso* isoleucine, *Lys* lysine, *Met-SO* methionine sulfoxide, *Phe* phenylalanine, *Pro* proline, *SDMA* symmetric dimethylarginine, *Thr* threonine, *Total DMA* total dimethylarginine, *Trp* tryptophan, *Tyr* tyrosine, *Val* valine.

Partial least square discriminant analysis (PLS-DA) revealed that the metabolomics profile in the vitreous of recovering FDEP eyes (n = 28) were significantly different from controls (n = 28) whether animals were raised under BEW (n = 14) (AUROC (test set, 1000 models) = 0.88; median *P* value = 0.047, paired *t* test*;* Supplementary Fig. S5) or SW (n = 14) (AUROC (test set, 1000 models) = 0.86; median *P* value = 0.04, paired *t *test; Supplementary Fig. S5) lights. Vitreous of recovering FDEP eyes displayed an increase in biogenic amines (i.e., dihydroxyphenylalanine (DOPA), ornithine, methionine sulfoxide (Met-SO), total dimethylarginine (total DMA) and amino acids (i.e., arginine, lysine and tryptophan), compared to control eyes (Fig. 6). BEW was specifically associated with additional increases in lipid (sphingolipids and glycerophospholipids), symmetric dimethylarginine (SDMA), glutamate and serotonin concentrations compared to control eyes. FDEP eyes reared under SW light exhibited a reduction in glycerophospholipids and increases in taurine, *trans*-4-Hydroxyproline (t4-OH-Pro) and various amino acids (i.e., tyrosine, proline, histidine, phenylalanine, alanine, threonine, valine and isoleucine) compared to controls eyes (Fig. 6). No predictive model was found to discriminate the retinas of control and recovering FDEP eyes (AUROC (test set) = 0.78; median *P* = 0.08).

### The vitreal and retinal metabolomic profiles of control and recovering FDEP eyes are dependent upon the spectral content of ambient light

**Figure 7:**
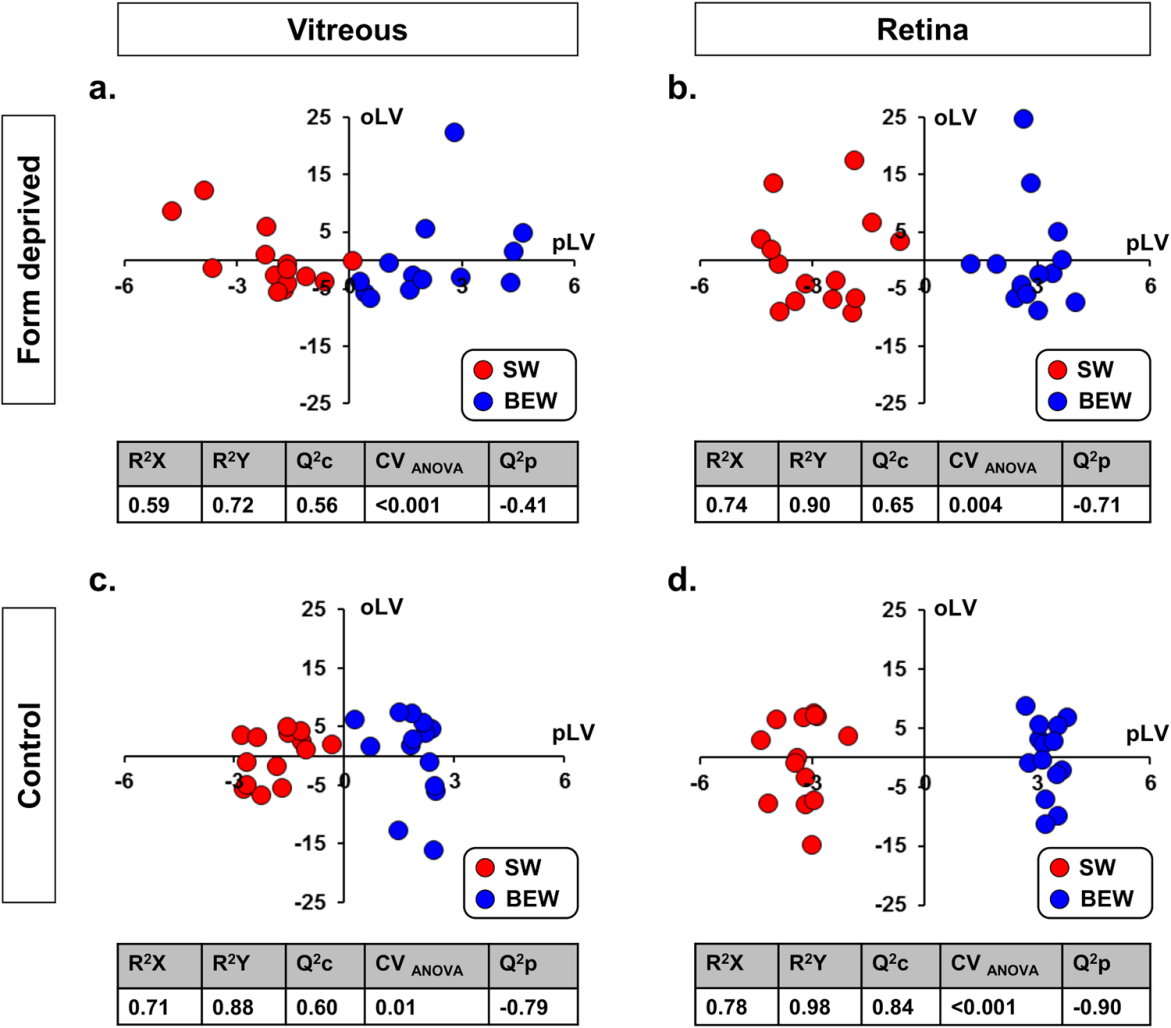
OPLS-DA on the metabolomic profiles of the vitreous and retina in recovering FDEP and control eyes. The metabolomic profiles were significantly different in the vitreous (**a**) and retina (**b**) of FDEP eyes of animals reared under SW or BEW light with Q^2^c scores of 0.56 and 0.65, respectively. Similarly, the metabolomic profiles were significantly different in the vitreous (**c**) and retina (**d**) of control eyes between animals reared under SW and BEW lights with Q^2^c scores of 0.60 and 0.84, respectively.

**Figure 8:**
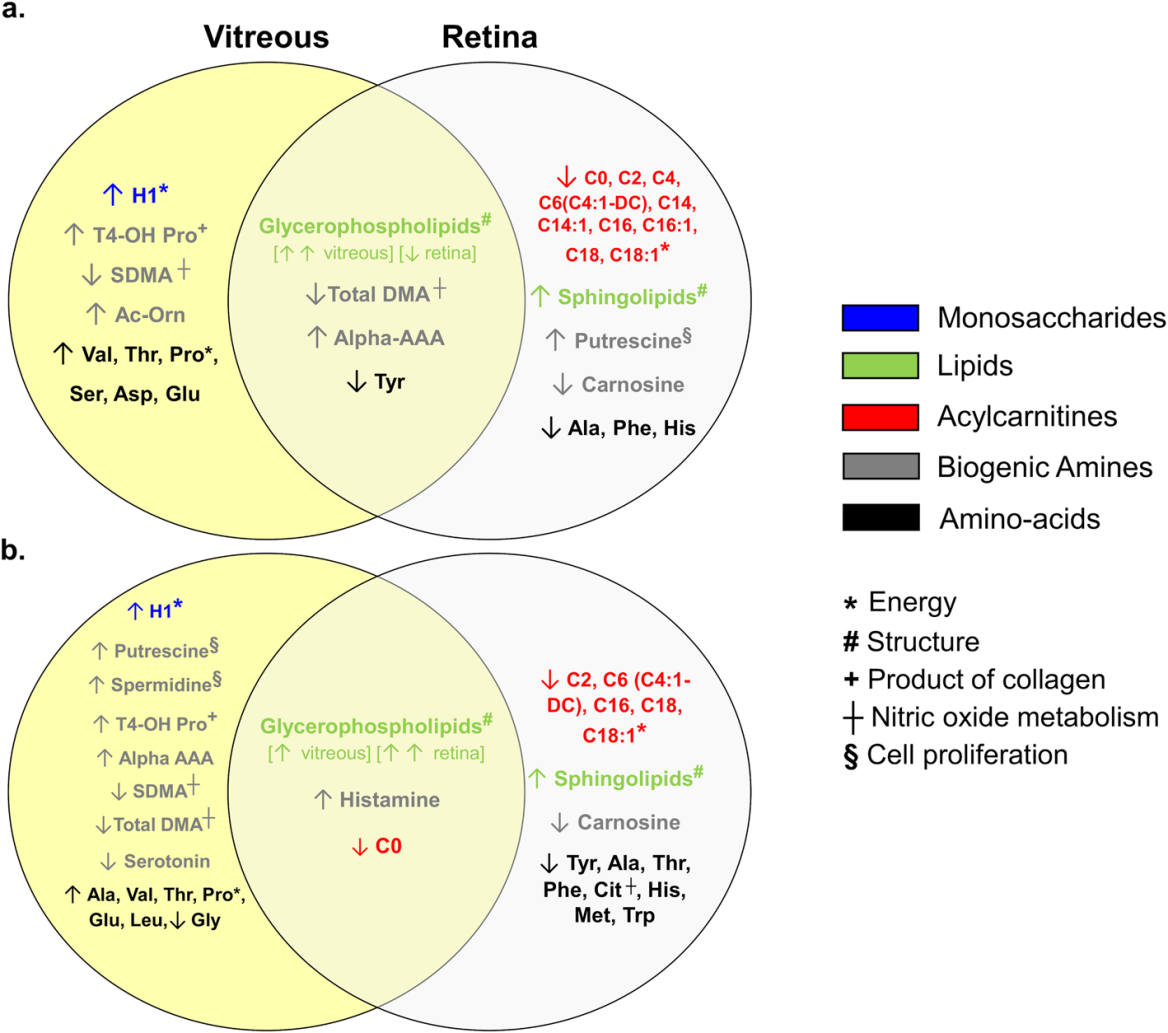
Venn diagrams summarizing metabolomic changes in the vitreous and retina of recovering FDEP (**a**) and control (**b**) eyes in animals exposed to BEW light compared to SW light. Compared to eyes reared under SW light, the metabolomic profiles within the FDEP and control eyes reared under BEW, involved an increase in vitreal H1, a reduction in retinal acylcarnitines, as well as increases in glycerophospholipids and sphingolipids in the vitreous and retina, respectively. Recovering FDEP eyes and control eyes exposed to BEW light also exhibited changes in biogenic amines (e.g., total and symmetric dimethylarginine (total DMA and SDMA), putrescine, spermidine, alpha aminodipic acid (alpha-AAA) and serotonin) and amino acids (e.g., alanine, valine, threonine, histidine and tyrosine) in the vitreous and retina. *Alpha-AAA* alpha-aminodipic acid, *Ala* alanine, *Asp* aspartate, *C0* carnitine, *C2* acetylcarnitine, *C4* butyrylcarnitine, *C6* hexanoylcarnitine, *C6* (*C4:1-DC*) hexanoylcarnitine (fumarylcarnitine), *C14* tetradecanoylcarnitine, *C14:1* tetradecenoylcarnitine, *C16* hexadecanoylcarnitine, *C18* octadecanoylcarnitine, *C18-1* octadecenoylcarnitine, *Cit* citrulline, *Glu* glutamate, *H1* sum of hexoses (including glucose), *His* histidine, *Iso* isoleucine, *Lys* lysine, *Met* methionine, *Phe* phenylalanine, *Pro* proline, *SDMA* symmetric dimethylarginine, *t4-OH Pro* *trans*-4-hydroxyproline, *Thr* threonine, *Total DMA* total dimethylarginine, *Trp* tryptophan, *Tyr* tyrosine, *Val* valine.

Orthogonal partial least squares discriminant analysis (OPLS-DA) revealed that the metabolomic profile in the vitreous and retinas of recovering FDEP eyes (n = 28) were highly dependent upon the spectral content of the light exposure [vitreous: Q^2^c: 0.56; *P* < 0.001, (Fig. 7a); retina: Q^2^c: 0.65; *P* = 0.004 (Fig. 7b)]. Similarly, the metabolomic profile in the vitreous and retina of control eyes were also dependent upon the light condition [vitreous: Q^2^c: 0.60; *P* = 0.01, (Fig. 7c); retina: Q^2^c: 0.84; *P* < 0.001, (Fig. 7d)]. Profiles within the FDEP and control eyes reared under BEW, involved an increase in vitreal monosaccharides (H1), a reduction in retinal acylcarnitines, as well as increases in glycerophospholipids and sphingolipids in the vitreous and retina, respectively. FDEP and control eyes exposed to BEW light also exhibited changes in biogenic amines (e.g., total dimethylarginine (total DMA) and SDMA, putrescine, spermidine, alpha aminodipic acid (Alpha-AAA) and serotonin) and amino acids (e.g., alanine, valine, threonine, histidine and tyrosine) in the vitreous and retina. Changes in ocular metabolite levels are detailed in the Venn diagrams for recovering FDEP (Fig. 8a) and control eyes (Fig. 8b). Raw metabolomics results are provided in Supplementary Data File 1.

## Discussion

In this study, we show that exposure to moderate intensities of blue-enriched white light can slow axial elongation, reduce aberrant ocular structural changes and accelerate recovery from increased axial ocular growth in a chicken model of form-deprivation myopia. Additionally, the spectral composition of ambient light modified the metabolomic profiles in both vitreous and retinas of control and recovering FDEP eyes. These strong spectrum-dependent signatures suggest that exposure to BEW light is associated with ocular changes in energy consumption and a remodeling of lipids and collagen.

The effects of narrow-band lights on ocular growth and refractive development have been evaluated in different animal models including non-human primates^39,41,42^, tree shrews^40^, fish^31,46^, Guinea pigs and chickens^22,35,47,48^. Our findings complement and add to earlier research showing a hyperopic shift in chickens exposed to narrow-band short wavelength and UV light^22,34,35^, an effect that is reversible when animals are reared with red light^22^. Amongst plausible explanations to these findings, ocular longitudinal chromatic aberration (LCA), which leads to wavelength defocus and higher refraction of short-wavelength light compared to long-wavelength light by ocular optics, was supported by many authors^35,49,50^. By virtue of LCA, under polychromatic (white) light as opposed to narrowband light, wavelength defocus produces an additional chromatic cue for the sign of defocus determined by the eye^51^. This notion is supported by the fact that emmetropization is more accurate under white light compared to monochromatic light^52,53^. It is plausible that the myopic, blue-shifted, chromatic defocus picked up under BEW light accelerated recovery from form-deprivation and led to a shorter axial length in control eyes. The latter statement is in agreement with recent evidence showing that, in chickens, axial ocular growth decreases with increasing S-cone contrast at low temporal frequency^54^. However, LCA is unlikely to be solely responsible for the large extent of deceleration in axial elongation under BEW light by the end of form-deprivation.

When it comes to ocular growth and refractive error development, the spectral response to light, as well as the validity of the LCA theory, are dependent upon experimental models used. For instance, eyes of infant rhesus macaques fitted with red transmitting spectacles, or raised under red light become hyperopic^39,41^, alike tree shrews exposed to steady or flickering red light^40,55^. Differences in the spectral response between chickens, tree shrews and rhesus macaques do not appear to be class-dependent, but may be due to protocol differences^53,56^, as guinea pigs (mammals lacking UV-sensitive cones) respond in a similar fashion to chickens^47,48^. Furthermore, Liu and colleagues reported monochromatic red light as a risk factor for the development of myopia in some, but not all, rhesus monkeys^42^. While recent evidence supports the modulation of cone opsin expression by form-deprivation and the spectral filtering of light (red spectacles) in chickens^57^, in humans, compared to emmetropes, myopes display a higher sensitivity of L-cones compared to M-cones and a lower sensitivity to low spatial frequency S-cones stimulation^29,30^. To date, the light sensitivity spectrum for ocular growth and refractive error development has yet to be established in humans and animal models. Elucidating inter-species differences in the spectral response to light would lead to a better understanding of the protective effects of light against myopia and allow the development of tailored indoor lighting regimens, preserving vision and promoting proper ocular development.

The retina in vertebrates encompasses three types of photoreceptors: rods, cones and intrinsically photosensitive retinal ganglion cells (ipRGCs). Light-induced neural signals from these photoreceptors, converge to regulate image- and non-image-forming responses. ipRGCs express a predominantly blue-sensitive photopigment melanopsin (OPN4). Two orthologues of melanopsin [Opn4m (mammalian-like melanopsin) and Opn4x (xenopus-like melanopsin)] have been identified in the chicken retina. Opn4m is expressed in a subset of retinal ganglion cells, while Opn4x is expressed in ganglion and horizontal cells^58^. The primary role of ipRGCs is to photoentrain the circadian timing system ^59^. In addition, ipRGCs directly regulate ocular dopamine secretion^60^, as a subset of dopaminergic retinal amacrine cells receive a tonic and persistent signal from ipRGCs influencing retinal light adaptation^61–63^. Consequently, the protective nature of BEW light against ocular growth could also be due to the increased intrinsic stimulation of ipRGCs and its effects on the retinal dopamine and melatonin cycles^64^.

While most changes in ocular refraction observed in animal models and humans can be explained by the axial elongation of the eye, aberrant corneal curvature and ACD have also been reported in multiple experimental models, but predominantly in highly myopic chickens^65,66^. Owing to the severity of axial elongation induced by our form-deprivation strategy, we also observe alterations in the anterior segment of FDEP eyes. These changes included an increase in ACD under both lighting conditions, albeit, to a lesser extent under BEW, and a thinning of the cornea only under SW light. The increase in ACD of FDEP eyes compared to controls was expected and only accounted for 19.8% and 30.3% of the overall axial elongation on D14 under BEW and SW lights, respectively. Conversely, corneal thinning have only recently been reported in form-deprivation models of chickens^67^, but is not unusual in humans^68^. Interestingly, anterior segment alterations during form-deprivation were not corrected (especially CCT under SW) during the 14 days recovery phase and were possibly epiphenomena of excessive ocular or posterior segment growth in an ordinarily growing orbit.

The choroid is a multifunctional structure, which plays key roles in retinal nutrition and other functions. It contains various tissue/cell types and has been proposed to play important roles in the visual regulation of ocular growth (for review^69^). Similarly to positive defocus, recovery from form-deprivation is associated with a rapid compensatory choroidal thickening, displacing the retina anteriorly, as a form of rapid compensation for the induced myopic defocus^70^. While it has already been reported that bright light (15,000 lux) induces choroidal thickening^71^, our histological findings show that choroid thickening in healthy and recovering FDEP eyes is also dependent upon the spectral composition of white light. Bearing in mind the limited sample size in our histological assessments, our findings are in agreement with findings from Rucker’s group showing that at an intermediate temporal frequency (5 Hz), the counter-phase, sinusoidal modulation of blue/yellow light reduced choroidal thinning in uncovered chickens eyes compared to red/green light^38^. Furthermore, Ostrin’s group recently showed that 1 h of narrow band blue light prevents choroidal thinning observed under 1 h of red light or darkness in humans^72^. On the other hand, when comparing FDEP eyes exposed to BEW to those exposed to SW light (Table 2), choroidal thickening (e.g., D14: + 0.011 mm; D22: + 0.073 mm) alone does not explain the deceleration in axial elongation observed during form-deprivation (D14: 0.60 mm), nor does it fully explain the acceleration in the recovery from form-deprivation (D22: 1.17 mm). Changes in axial length under BEW light are likely due to a combination of choroidal thickening and a reduction in ocular growth rate.

Understanding the anatomical-physiological conditions and metabolic status of tissues and organs allows for insight into pathogenic mechanisms underlying various systemic and ocular conditions^73^. In addition to structural changes in the posterior and anterior ocular segments, we report that BEW light induced metabolomic variations in the vitreous and retina of recovering FDEP and healthy chicken eyes. Changes included a modulation of energetic substrates and a deep phospholipid remodeling within the vitreous and retina. Myopia progression has previously been associated with an inverse relationship between glucose accumulation and decreases in lipids content, in FDEP guinea pig eyes^74^, therefore, increases in H1 and decreases of acylcarnitines, reflecting an increase of oxidative energy production, may have contributed to the faster recovery from form-deprivation in eyes exposed to BEW light compared to eyes exposed to SW light. Proline, which was increased in the vitreous under BEW light, in both controls and recovering FDEP eyes, is also an energetic substrate playing a preponderant role in the retina^75^. Furthermore, BEW light induced a decrease in carnosine concentrations in both recovering FDEP and control eyes. Carnosine is an abundant neuroprotective dipeptide in the retina that regulates glycolysis and oxidative energy metabolism and could therefore be involved in the modifications of energy substrates observed in the metabolomic profiles^76^. The global content of glycerophospholipids and sphingolipids was strongly altered both in the vitreous and retinas of animals exposed to BEW light compared to SW light. These lipids being the major component of cell membranes, these changes are likely to indirectly reflect the global cell content remodelling in the choroid and retina. On the other hand, increases in retinal glycerophospholipids and more specifically phosphatidylcholines, have been associated with light stress in rats^77^. We do not believe that changes in glycerophospholipids reported in our study are solely related to retinal stress by moderate levels of BEW light since (1) increases in glycerophospholipids were observed in recovering FDEP eyes in comparison with control eyes exposed to the same levels of BEW light (Fig. 6) and (2) glycerophospholipids (phosphatidylcholines) were decreased in the retinas of recovering FDEP eyes exposed to BEW light in comparison to retinas exposed to SW light (Fig. 8).

SDMA is derived from intra-nuclear methylation of arginine residuals in proteins and is released by protein and cell turnover. SDMA being an inhibitor of endothelial nitric oxide synthase (eNOS) activity^78^, its reduced content in the vitreous of both control and recovering FDEP eyes under BEW light, may increase NO production. NO, released from the choroid or retina, is involved in choroid thickening and ocular growth inhibition^79–81^. Similarly, increase in histamine, as observed in control eyes exposed to BEW light, have been reported to increase blood flow and vessel diameters of the choroid in humans^82^, a phenomenon that is also reported prior to choroidal expansion and filling of the choroidal lymphatic lacunae in chickens^83^. Increases in *trans*-4-Hydroxyproline (a degradation product of collagen in extracellular matrices reflecting collagen turnover) and the modified content of polyamines putrescine and spermidine (involved in the regulation of division, differentiation and maturation of cells), could also be related with the increased choroidal thickening observed under BEW light. Conversely, citrulline, reported in higher concentrations in the aqueous of patients with high myopia, is reduced under BEW light^84^; and valine and threonine, reduced in the retina of FDEP eyes of Guinea pig^74^, were found to be increased in the vitreous (not the retina) of recovering FDEP eyes exposed to BEW light. Finally, exposure to BEW increased retinal and vitreal concentrations of alpha-aminoadipic acid. Interestingly, intra-vitreal injection of DL-alpha-aminoadipic acid can suppress the development of form-deprivation myopia in Guinea pigs^85^.

Retinal dopamine synthesis is well known to be reduced during the development of form-deprivation myopia in chicks^86^, while retinal dopamine and its metabolite 3,4-dihydroxyphenylacetic acid (DOPAC) have been reported to rise early after diffuser-removal and to be correlated with recovery from myopia^87^. Our metabolomic analyses show higher vitreal concentrations of DOPA in the recovering FDEP eyes compared to control eyes, independently of the lighting condition. These findings are consistent with a recent report by Wang and colleagues showing that retinal dopamine and vitreal DOPAC levels increase under blue or red lights in chickens^34^. However, no changes were found in dopamine levels in the recovering FDEP eyes, despite a significant drop in tyrosine, a precursor of dopamine, in control and recovering FDEP eyes exposed to BEW light. It’s plausible that a rise in dopamine may have occurred at an earlier time-point during the recovery from form-deprivation. On the other hand, serotonin levels were increased in the vitreous of recovering FDEP eyes and decreased in the vitreous of control eyes exposed to BEW. Serotonin is a precursor of melatonin that modulates the response of the circadian system to light. Collectively, the literature suggests that manipulations of the ocular serotoninergic system can affect eye growth, to date, however, the exact process of how that might occur remains uncharacterized. For instance, pharmacologic depletion of serotonergic neurons blocks the development of form-deprivation myopia in chicks^88^, while serotonergic antagonists inhibit the development of lens-induced myopia in chicks^89^. Serotonin has also been shown to induce vasoconstriction in the choroidal blood vessels of rats^90^. The modulation of the serotoninergic system via light may be associated with changes in choroidal thickness.

Our study has a few limitations. First, although SW and BEW lights were closely matched for illuminance, an irradiance difference of 14.89 µW/cm^2^ was unavoidable between conditions (Table 1). Whether such a minor difference in irradiance could lead to changes in the metabolomic profile is unclear. Second, the posterior borders of thickened choroids on D22 and D28 were occasionally not clearly visible on posterior segment OCT scans (total of 5 animals: SW, n = 2; BEW, n = 3). In these cases, measurements were excluded, which may have reduced the statistical power to detect the thicker choroid under BEW compared to SW lights on D22 and D28. Third, while choroidal thickness results obtained in vivo using OCT confirm histological findings in control eyes, they do not show a significant increase in choroidal thickness in the FDEP eyes exposed to BEW light compared to SW light. This incoherence between choroid assessment methods can be due to (1) increased variability in the OCT assessments of FDEP eyes exposed to SW light; (2) differences in the average choroidal thickness of samples investigated using the 2 methods and/or (3) the fact that histological measurements took place on D29 while the last OCT measurement occurred on D28. OCT and histological assessments of choroidal thickness were, however, significantly correlated (n = 20, Pearson correlation; *R* = 0.65, *P* = 0.002). Fourth, even though we report a significant impact of the spectral composition of light on ocular metabolomic signatures, the precise interpretation of such changes in metabolite concentrations through our analysis remains challenging since these changes may have multiple cellular and metabolic origins^91^. In addition, while our study highlights metabolomic changes specifically related to the recovery from form-deprivation under distinct lighting conditions, further studies are needed to also explore such changes during the development of myopia under different lighting conditions. Finally, findings from this study may have limited direct clinical applications given the anatomic, physiologic and metabolomic ocular differences between birds and humans. Nonetheless, the overall spectral sensitivity to light is closely similar between chickens and humans, with differences predominantly residing in the UV region (< 400 nm) of the light spectrum^34,92^, and despite genetic and evolutionary differences between birds and mammals, core metabolic functions remain similar between the two classes of vertebrates^93^.

Without adequate interventions, myopia is projected to affect 50% of the world population by 2050, becoming the leading cause of irreversible blindness^14^. Today, increasing outdoor time remains intricate and exposing children to narrow-band lights could be detrimental for visual performance^94^. Our findings show that, at moderate light levels (~ 230 lux) similar to those experienced at standard households, blue-enriched white light can slow aberrant axial elongation and accelerate recovery from form-deprivation in a chicken model. Moreover, from a mechanistic facet, we show that the chromaticity of ambient light can modify the metabolomic profile of the vitreous and retinas in healthy and myopic eyes. In no way are we suggesting that our findings are directly applicable in humans, yet, here, we provide strong evidence that the spectral tailoring of indoor white light may offer an effective and non-invasive light therapy strategy to tackle myopia as a leading health and socio-economic burden in a further, indoor-centered, digitized world.

## Methods

### Study design

The aim of this controlled animal experiment was to investigate the impact of ambient and moderate light levels of BEW light on ocular growth and metabolomics profiles in a chicken model of form-deprivation myopia. To achieve this aim, 36 newly hatched chicks (Lohmann Brown, 18 males) were raised for 29 days on a 12 h/12 h light dark cycle (7 am–7 pm) in a light-tight cuboid enclosure [69 × 46 × 38 cm (Length × Width × Height)] equipped with tunable light emitting diode (LED) lighting systems and surrounded with accommodative cues that included a wallpaper of black and white vertical stripes (spatial resolution range: 0.01—0.59 cycle per degree) in addition to feeding and drinking trays. Temperature was maintained between 28 and 32 °C via a heating system equipped with a thermostat. Animals had ad libitum access to feed of the same brand and water. Light patterns and temperature were monitored across each experimental batch using loggers (Data-loggers, Mindset, UK). Sample size was calculated prior to the start of the study based on preliminary pilot data. For α = 0.05, a sample size of 18 chicks per group was required to provide a statistical power of 90% for the detection of an effect size of 1.1 mm in the primary outcome measure of the study (i.e., axial length) between FDEP eyes of the study groups, by the end of the form-deprivation period (D14)^95^. Investigators were blinded to the experimental condition and study eyes during data processing and analysis. The study was carried out in compliance with the ARRIVE guidelines (https://arriveguidelines.org). Animals used in this study were treated in accordance with the Association of Research in Vision and Ophthalmology Statement for Use of Animals in Ophthalmic and Vision Research. Ethical approval was obtained from the Animal Care and Use Committee, Singhealth, Singapore, AAALAC accredited (IACUC 2015/SHS/1057).

### Lighting conditions

Animals were randomly separated into four batches of 9 animals each. Batches 2 and 4 (group 1, n = 18, 9 males) were reared under 3900 K SW LED light (2NFLS-NW LED, Super Bright LED, Inc, MO, USA) (Fig. 1) while batches 1 and 3 (group 2, n = 18, 9 males) were reared under 9700 K BEW light (2NFLS-CW LED, Super Bright LED, Inc, MO, USA). Light levels under both conditions were of similar illuminance and photon density (Table 1) and were monitored bi-weekly using calibrated radiometer and spectroradiometer (ILT5000 and ILT950, International Light Technologies, Peabody, MA, USA).

### Form-deprivation

Form-deprivation was induced monocularly in each animal using a customized 3D printed frosted diffuser. Diffusers were mounted randomly onto one of the chick’s eyes using Velcro with one side glued to the diffuser and the other glued to the down surrounding the animal’s eye. The fellow eye was left uncovered and served as control. Diffusers were inspected for cleanliness and positioning daily by a study team member. Form-deprivation took place from Day 1 (D1) to D14 post-hatching. On D14 diffusers were removed and recovery from the intervention was assessed between D14 and D28. Diffusers attenuated light levels and altered the spectral composition of light reaching the cornea in a similar fashion for both SW and BEW lights (Table 1, Fig. 1).

### Ocular measurements in vivo

Weighing and ophthalmic measurements were performed on alert and gently handled chicks on D1, D7, D14, D22 and D28 in a dimly lit experimental room (< 1 lux). In vivo measurements consisted of ocular AL, retinal and choroidal thicknesses as well as ACD and CCT (Fig. 2). AL was assessed using ultrasonography (PacScan, Sonomed, NY, USA). The sampling frequency of the ultrasound was 10 MHz and AL was defined as the distance between the echo spike corresponding to the anterior surface of the cornea and most anterior spike originating from the retina. The median of 7–10 measurements per eye was calculated for each animal.

Choroidal and retinal thicknesses at the posterior pole of the eye were measured in alert, hand-held chicks, using optical coherence tomography (Spectralis OCT, Heidelberg Engineering, Germany) following the protocol adopted by Lan et al.^71^. The axial and lateral resolutions of the system were 3.87 μm and 5.42 μm, respectively. During the measurements, the operator gently handled the chicken and positioned its head in alignment with the OCT’s camera lens so that the infra-red laser beam entered the eye through the center of the pupil. Once proper alignment and centration of the pupil was refined by the operator, multiple OCT single scans of the posterior pole (30°) were captured for each eye. Subsequently, scans in which the pupil was properly centered (within ± 100 µm from the horizontal line) and the borders of the individual fundal layers were clearly visible (Supplementary Fig. S6) were used for further analysis. Choroidal (n = 3 per image) and retinal thickness (n = 3 per image) measurements were averaged from at least 2 eligible scans per eye. Measurements were performed manually by the first author RPN using the Heidelberg Eye Explorer software (Heidelberg Engineering, Germany) and after completion of all experimental procedures of the study. RPN was blinded to both the eye (form-deprived or control) and study group (BEW or SW) when performing the measurements. Choroidal thickness was defined as the distance between the inner border of the sclera and the outer border of the RPE^96^. The segmentation of the choroid was done manually. Retinal thickness was defined as the distance between the inner limiting membrane and Bruch’s membrane. The segmentation of the retina was performed automatically by the Heidelberg Eye Explorer software (Heidelberg Engineering, Germany) (Supplementary Fig. S7). The average coefficient of variation for repeated choroidal measurements was 6.5 ± 5.2% while the average coefficient of variation for repeated retinal measurements was 3.1 ± 1.7%.

Anterior segment features (CCT and ACD) were imaged using anterior segment OCT (RTvue, Optovue, CA, USA). During the imaging procedure, the operator gently handled the alert chicken and positioned its head in alignment with the OCT’s camera lens so that the infra-red laser beam entered the eye through the center of the pupil. Once proper alignment and centration of the pupil was refined by the operator, multiple scans of the anterior segment (pachymetry mode) were captured for each eye. Scans in which the anterior segment was fully visible were used for further analysis. CCT was defined as the thickness of the cornea at its central portion and ACD was defined as the distance between the central most posterior layer of the cornea and the central most anterior layer of the lens (Supplementary Fig. S8). Measurements (one measurement per eye) of ACD and CCT were performed by a lab technician using the inbuilt RTvue software after completion of all experimental procedures of the study. The lab technician was blinded to both the eye (form-deprived or control) and study group (BEW or SW) when performing the measurements.

All measurements took place between 12 pm and 5 pm and animals were evaluated in a random order to minimize any circadian impact on the study outcomes. A lid retractor was required on D1 of the experimental protocol in some animals that were unable to keep their eyelids open.

### Ocular measurements ex vivo

On D29, chicks were euthanized after heavy sedation (Ketamin 0.2 ml/kg–Xylazin 0.1 ml/kg) using an overdose of intra-cardiac sodium pentobarbitone. Euthanasia took place in a dimly lit room between 10 am and 5 pm.

#### Histological preparations and measurements

Histological analyses were performed in the eyes of 4 animals from each group. To improve the statistical power of our analyses we increased the sample size of the SW group by adding histological assessments from 2 animals reared under SW light that underwent the same experimental protocol. Whole eyes were enucleated and immersed in 10% Neutral Buffered Formalin (NBF) (Leica-Surgipath, Leica Biosystems Richmond, IL, USA) to fix the tissues for bilateral histological examinations of the choroid, retina and sclera. Eyes were then dissected at the ora serrata and anterior segment and vitreous were removed. The remaining posterior segment was subjected to dehydration in increasing concentration of ethanol, clearance in xylene, and embedding in paraffin (Leica-Surgipath, Leica Biosystems Richmond, IL, USA)^97^. Five-micron sections were cut with a rotary microtome (RM2255, Leica Biosystems Nussloch GmbH, Germany) and collected on POLYSINETM microscope glass slides (Gerhard Menzel, Thermo Fisher Scientific, Newington, NH, USA). The sections were dried in an oven of 37 °C for at least 24 h. Prior to histological examination, sections were heated on a 60 °C hot plate, deparaffinized in xylene and rehydrated in decreasing concentration of ethanol^97^. A standard procedure for Hematoxylin and Eosin (H&E) staining was performed and sections were mounted with mounting medium (Fisher Scientific Permount Mounting Medium SP15-500). A light microscope (Axioplan 2; Carl Zeiss Meditec GmbH, Oberkochen, Germany) was used to examine the slides of the posterior pole and images were captured. Thicknesses of the choroid, retina and sclera were manually measured at the posterior pole using Axioplan 2’s software ‘Distance’ tool.

#### Metabolomics

Targeted quantitative metabolomic analysis was carried out on the eyes of 28 animals using the Biocrates Absolute IDQ p180 kit (Biocrates Life Sciences AG, Innsbruck, Austria). This kit uses mass spectrometry (QTRAP 5500, SCIEX, Villebon-sur-Yvette, France) to quantify up to 188 different endogenous molecules including: free carnitine (C0), 39 acylcarnitines (C), the sum of hexoses (H1), 21 amino acids, 21 biogenic amines and 105 lipids. Four different classes of lipids are detected by the kits: 14 lysophosphatidylcholines (lysoPC), 38 diacyl-phosphatidylcholines (PC aa), 38 acyl-alkyl-phosphatidylcholines (PC ae) and 15 sphingomyelins (SM). Additional details on the full list of individual metabolites are available at http://www.biocrates.com/products/research-products/absoluteidq-p180-kit^98^. Carnitine, acylcarnitines, lipids and hexoses were investigated using flow injection analysis coupled with tandem mass spectrometry (FIA-MS/MS). Amino acids and biogenic amines were separated using liquid chromatography (LC) before quantitation with mass spectrometry. All reagents were of LC–MS grade and purchased from VWR (Fontenay-sous-Bois, France) and Merck (Molsheim, France). Sample preparation and analysis were performed following the Kit User Manual using the procedure described elsewhere^98^. The metabolomic analyses were directly performed on 10 μl of the supernatant of vitreous humor obtained after centrifugation (2000*g* × 5 min at 4 °C) and conserved at − 80 °C, whereas a metabolite extraction was performed for retinas in a cold solution of phosphate-buffered saline (PBS; 15 μl) and methanol (85 µl). Homogenization was performed using a Precellys homogenizer (Bertin Technologies, Montigny-le-Bretonneux, France) kept in a room at + 4 °C. The supernatant (retinal extract) was recovered after centrifuging the homogenate (10,000*g* × 5 min at 4 °C) and stored at − 80 °C until metabolomic analysis^99^.

### Statistical analysis

Weight, AL, ACD, CCT and thicknesses of the retina and choroid (assessed using OCT) were compared between eyes within the same group (e.g., within group 1—form-deprived eyes vs*.* control eyes) and across groups (e.g., form-deprived eyes—group 1 vs. group 2) during and after form-deprivation using a two-way repeated measures analysis of variance with day and group or day and eye as within- and between-subject factors, respectively. For those comparisons in which the omnibus test reached statistical significance, pairwise multiple comparison procedures were performed using the Holm-Sidak method. The threshold for significance for all statistical tests was set at α = 0.05 and Sidak correction was applied for all post hoc pairwise comparison. Comparison of histological choroidal, retinal and scleral thicknesses were performed using two-sided, paired (eye comparison) or unpaired (group comparison) Student’s *t* tests. Statistics and plots were performed using SigmaPlot Version 14.0 (Systat Software, San Jose, CA, USA). Data are represented in the text and figures as average ± SD.

#### Multivariate analysis of metabolomic data

Prior to statistical analyses on the metabolomic profile, raw data were examined to exclude metabolites having more than 20% of concentration values below the lower limit of quantitation (LLOQ) or above the upper limit of quantitation (ULOQ). Unsupervised analysis was carried out using principal component analysis (PCA). PCA allows detection of similar samples grouping together in the space determined by the principal components. Atypical samples appear far from the group formed by the majority of samples and can also be spotted by parameters like Hotelling’s T2 statistics. Atypical samples could be labelled as outliers and eliminated from further univariate and supervised multivariate statistical analyses after data and metadata examination. No samples were labeled as atypical in this study.

#### Multivariate statistical analysis for independent samples

Supervised statistical analyses were carried out using OPLS-DA. In a supervised analysis based on projection methods like OPLS-DA new variables called latent variables (LV) are found. These LV are linear combinations of the metabolites and the correlation with the response variable is maximized for the first LV, called predictive LV or pLV. The second LV (oLV) is orthogonal to pLV and so it’s not correlated to the response variable. In order to minimize overfitting of supervised models and considering limited sample size, models with only two LV (i.e., pLV and one oLV) were built. Overfitting in OPLS-DA models were evaluated by cross-validation using cross-validated R^2^Y (Q^2^Ycv or goodness of prediction), cross-validated analysis of variance (CV-ANOVA) test, and the goodness of prediction of models obtained by permuting the elements of the response variable while keeping the metabolite matrix unchanged (Q^2^p). Models with a low degree of over-fitting are characterized by Q^2^c > 0.5, negative Q^2^p and are significantly more discriminant than the null model (CV-ANOVA P < 0.05). PCA and OPLS-DA for independent samples were performed in SIMCA-P v14.1 software (SIMCA, Umetrics, Sweden).

#### Multivariate statistical analysis for paired samples

SIMCA-P, as many other available software, can’t handle paired samples so for the analysis of retinas and vitreous from both eyes of the same chick (i.e., paired samples) we used the R package mixOmics [R v.3.6.3: A Language and Environment for Statistical Computing (R Core Team, Vienna, Austria)]. This package performs PCA and PLS-DA analysis for paired samples using the same principle in the construction of principal components and latent variables. To validate supervised PLS-DA models obtained with mixOmics we used the training-test set strategy. We divided all samples in 18 (~ 2/3) and 10 samples (~ 1/3) allocated to the training and test sets, respectively. We constructed 1000 supervised PLS-DA models by permuting samples between the training and test set. Predictive capabilities of PLS-DA models built with the training set were measured in the test set using the area under the receiver operating characteristic curve (AUROC) and its associated p-value comparing PLS-DA model to the random model (i.e., AUROC = 0.5). Predictive capability of PLS-DA model obtained with all samples was considered as satisfactory when median AUROC was at least of 0.8 and its associated *P* < 0.05.

#### Univariate analysis of metabolomic data

Metabolite concentrations were log-transformed before performing Student’s *t* test between groups and Student’s *t* test for comparing paired samples. To correct for risk type I inflation due to test multiplicity, Benjamini–Hochberg correction was applied to keep false discovery rate under 10%. Univariate analyses were performed in Excel.

## Supplementary Information

Supplementary Data 1.

Supplementary Figures.

## Data Availability

The data that support the findings of this study are available from the corresponding authors upon reasonable request.
