# Supplementary material for: Ocular growth and metabolomics are dependent upon the spectral content of ambient white light
Source: Sci Rep. 2021 Apr 7;11:7586. doi: 10.1038/s41598-021-87201-2 (PMC8026599; doi:10.1038/s41598-021-87201-2)
Supplement: Supplementary file 2 — Supplementary Figures. [file 41598_2021_87201_MOESM2_ESM.pdf]

Ocular growth and metabolomics are dependent upon the spectral content of ambient white light

Raymond P. Najjar[1,2*], Juan Manuel Chao De La Barca[3,4], Veluchamy A. Barathi[1,2,5], Candice Ho Ee Hua[1], Jing Zhan Lock[1], Arumugam R. Muralidharan[1], Royston K.Y. Tan[6], Chetna Dhand[1,7], Rajamani Lakshminarayanan[1], Pascal Reynier[3,4], Dan Milea[1,2,8*]

[1] Singapore Eye Research Institute, Singapore

[2] The Ophthalmology and Visual Sciences ACP, Duke-NUS Medical School, Singapore

[3] Département de Biochimie et Génétique, Centre Hospitalier Universitaire d'Angers, Angers, France

[4] Unité Mixte de Recherche MITOVASC, CNRS 6015, INSERM U1083, Université d'Angers, Angers, France

[5] Department of Ophthalmology, Yong Loo Lin School of Medicine, National University of Singapore, Singapore

[6] Department of Ocular Bio-Engineering, National University of Singapore, Singapore

[7] CSIR-Advanced Materials and Processes Research Institute, Hoshangabad Road, Bhopal, India 462026

[8] Singapore National Eye Center, Singapore

[*] To whom correspondence should be addressed

Supplementary Figures

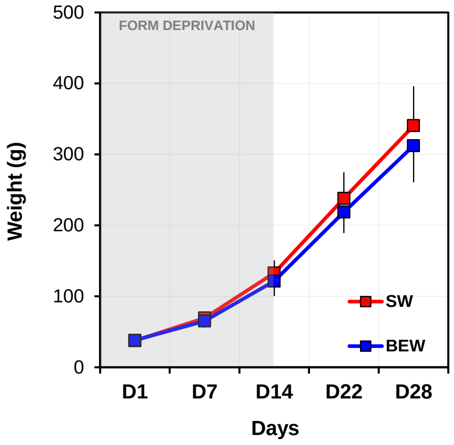

Supplementary Figure S1. Average weight of groups raised under SW (n = 18) or BEW (n = 18) light, across the experimental period. Weights were not different between groups on D1, D7, D14, D22 and D28 of the protocol ($P > 0.05$). Data are represented as average ± SD.

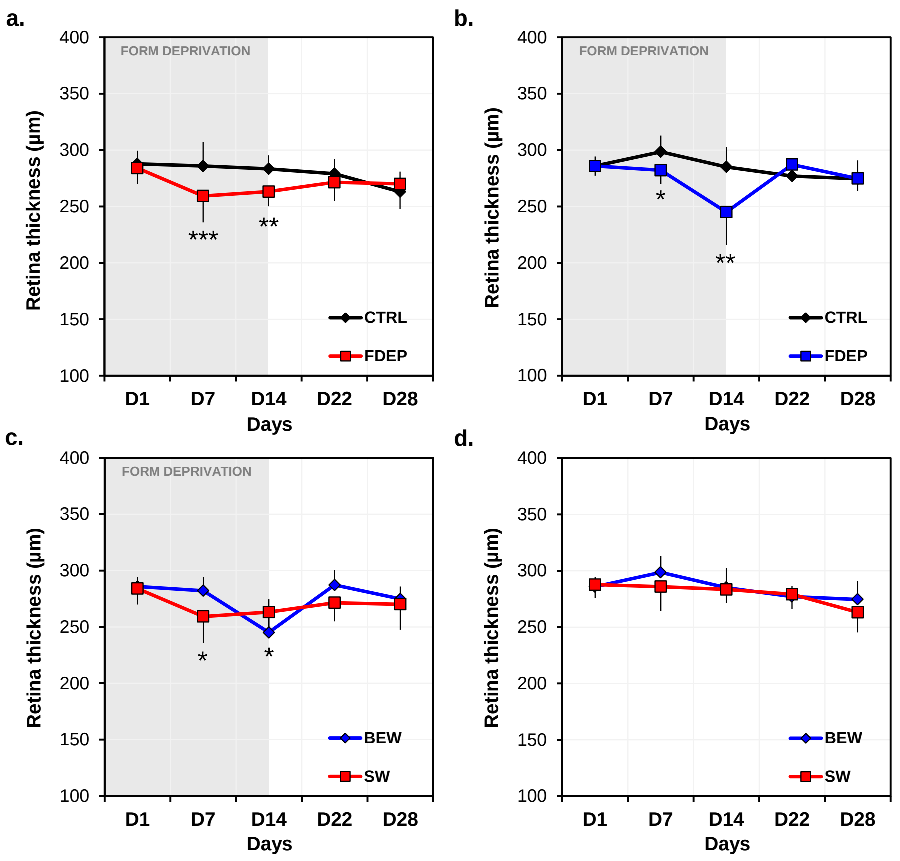

Supplementary Figure S2. Retinal thickness of the FDEP and control eyes of animals reared under BEW (n = 18) or SW (n = 18) lights. Retina thicknesses were reduced by form-deprivation (D7 and D14) under SW (a) and BEW (b) lights. Overall, retina thicknesses of FDEP (c) and control (d) eyes were not different between groups. Data are represented as average ± SD. Post hoc pairwise comparison significance: *: $P < 0.05$; **: $P < 0.01$; ***: $P < 0.001$.

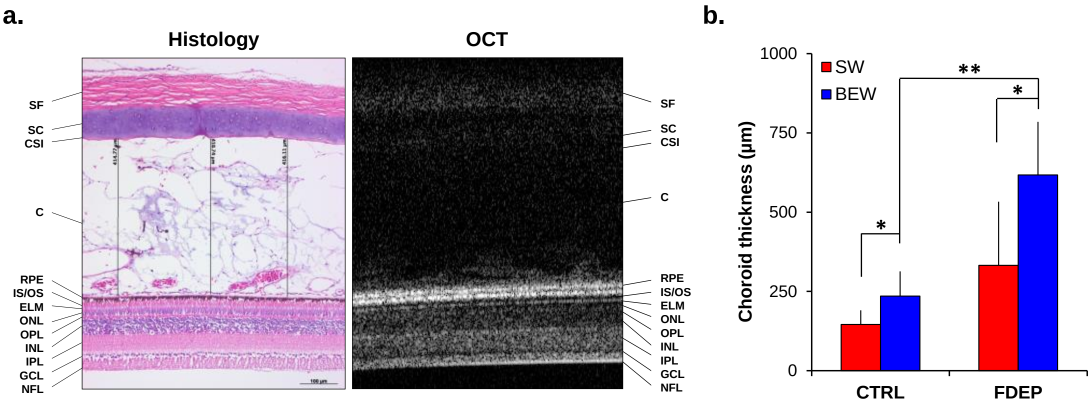

Supplementary Figure S3. Histological assessment of choroidal thickness. a. Histological cut of a chicken's posterior pole originating from 1 representative recovering FDEP eye (D29) reared under BEW light, compared to an OCT image of the posterior pole performed in the same animal (D28). **b.** Average choroid thicknesses measured on histological cuts showing increased choroid thickness in recovering FDEP and control eyes of animals raised under BEW light (n = 4) compared to SW light (n = 6) ($P < 0.05$). The choroid of recovering FDEP eyes was thicker compared to control eyes in both groups but only reached statistical significance in animals reared under BEW light ($P = 0.008$). Data are represented as average ± SD. T-test significance: *: $P < 0.05$; **: $P < 0.01$. **Abbreviations:** C: Choroid; CSI: Chorio-scleral interface; CTRL: Control eyes; ELM: External limiting membrane; FDEP: Form deprived eyes (recovering); GCL: Ganglion cell layer; INL: Inner nuclear layer; IPL: Inner plexiform layer; IS/IO: Inner and outer segments of the photoreceptors; NFL: Nerve fiber layer; OCT: Optical coherence tomography; ONL: Outer nuclear layer; OPL: Outer plexiform layer; RPE: Retinal pigment epithelium; SC: Cartilaginous sclera; SF: Fibrous sclera.

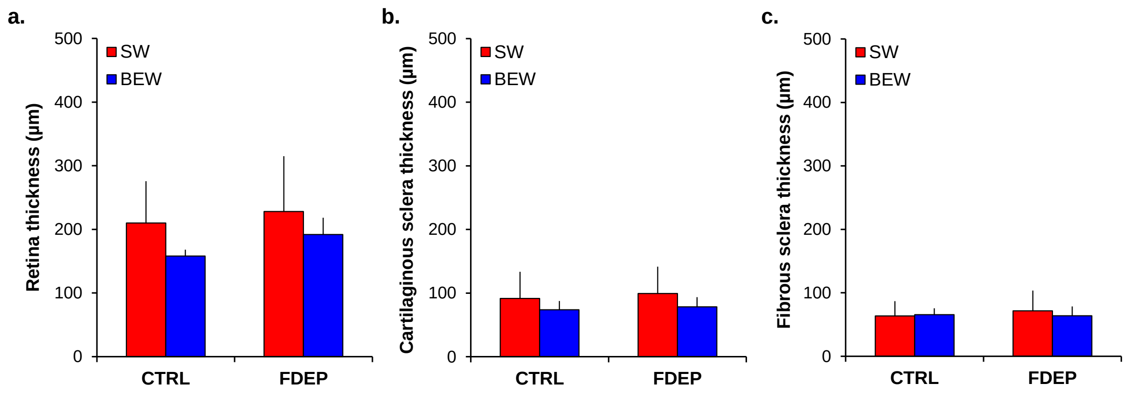

Supplementary Figure S4. Histological assessment of retinal and scleral thicknesses in control and recovering FDEP eyes. a. Average retina thicknesses were not different between groups or eyes. Average thicknesses of the cartilaginous **(b)** and fibrous **(c)** layers of the sclera were not different between groups and eyes. **Abbreviations:** CTRL: Control eyes; FDEP: Form deprived eyes (recovering). Data are represented as average ± SD.

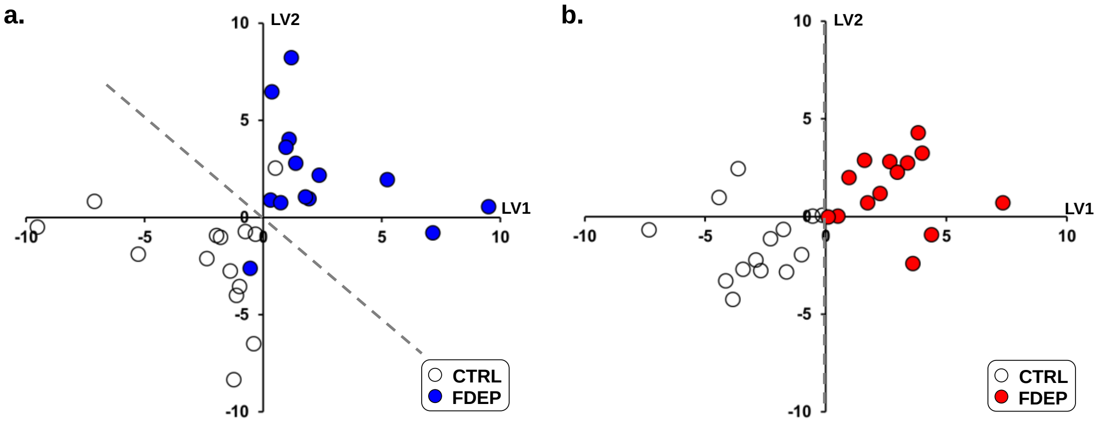

Supplementary Figure S5. PLS-DA showing distinct metabolomic profiles in the vitreous of recovering FDEP and control eyes under BEW (a) and SW (b) lighting environments.

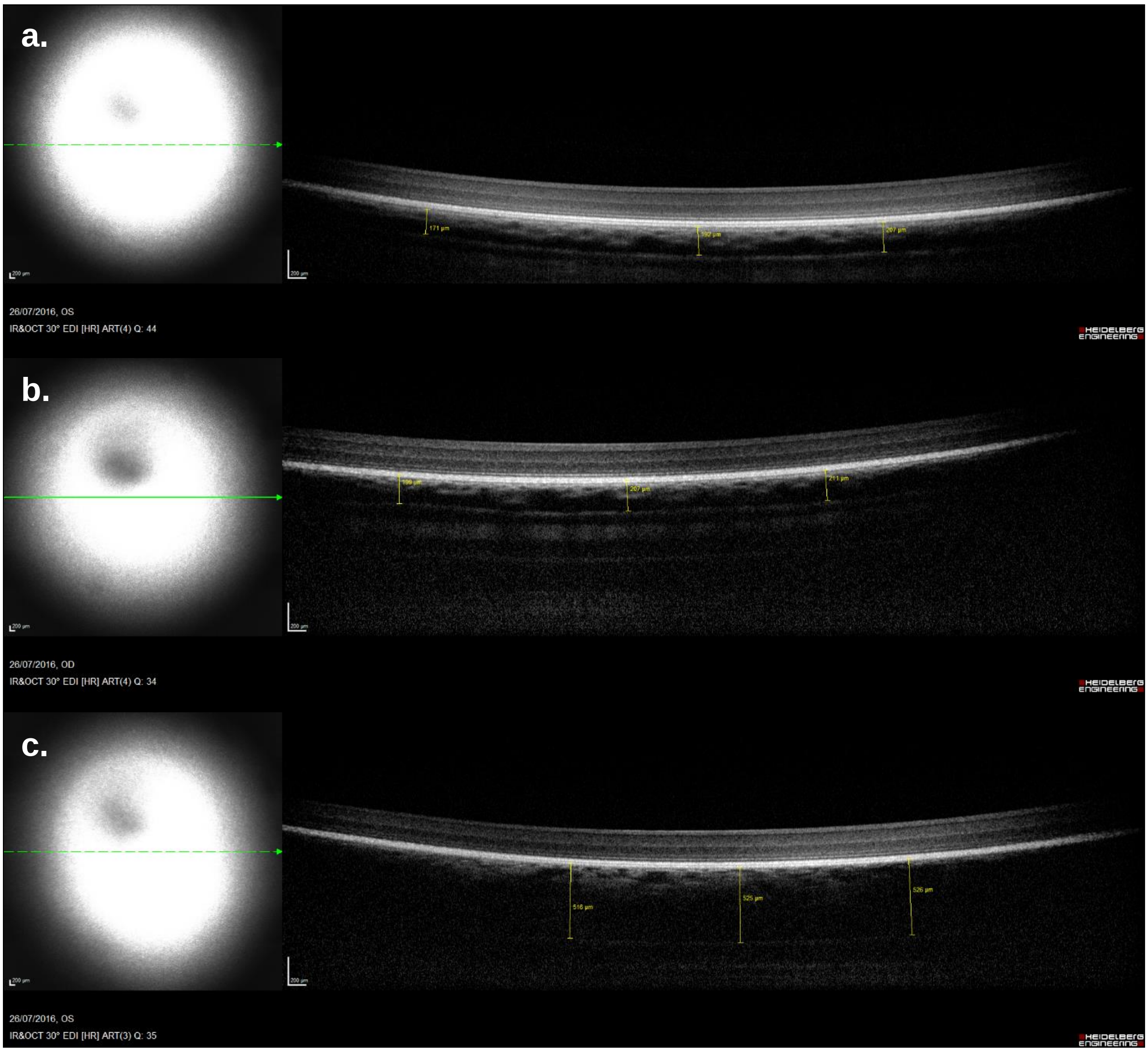

Supplementary Figure S6. Representative examples of posterior segment OCT scans and measurements of the choroidal thickness in the control eye of one chicken exposed to BEW on D28 (a) and in the control (b) and recovering FDEP (c) eyes of another chicken exposed to BEW light on D28.

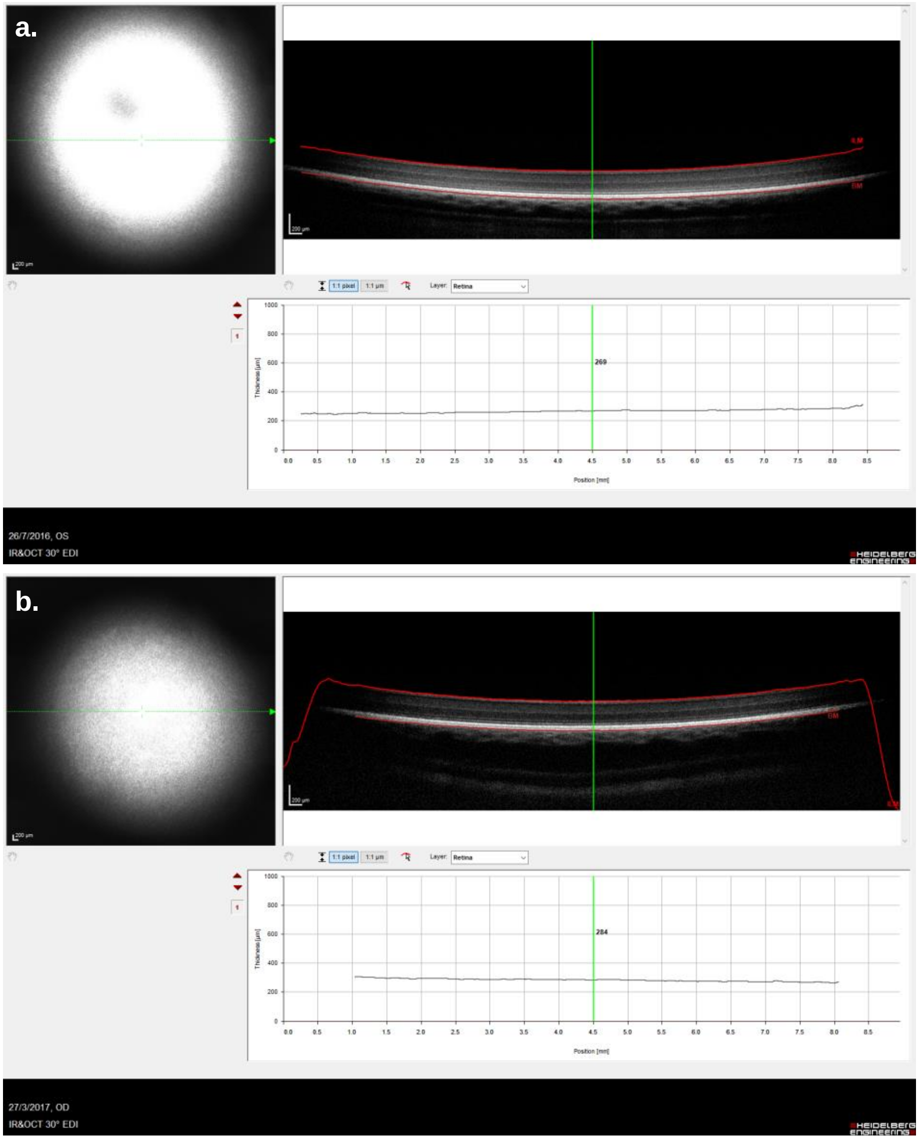

Supplementary Figure S7. Representative examples of posterior segment OCT scans and automatic segmentation and measurements of the retinal thickness in the control eye (a) and recovering FDEP eye (b) of two chickens. Both chickens were exposed to SW light and scans were taken on D28. Three measurements of the retinal thickness were taken: one at the central position of the scan (here 4.5 mm) and two at 1 to 1.5 mm around the center (here 3mm and 6mm).

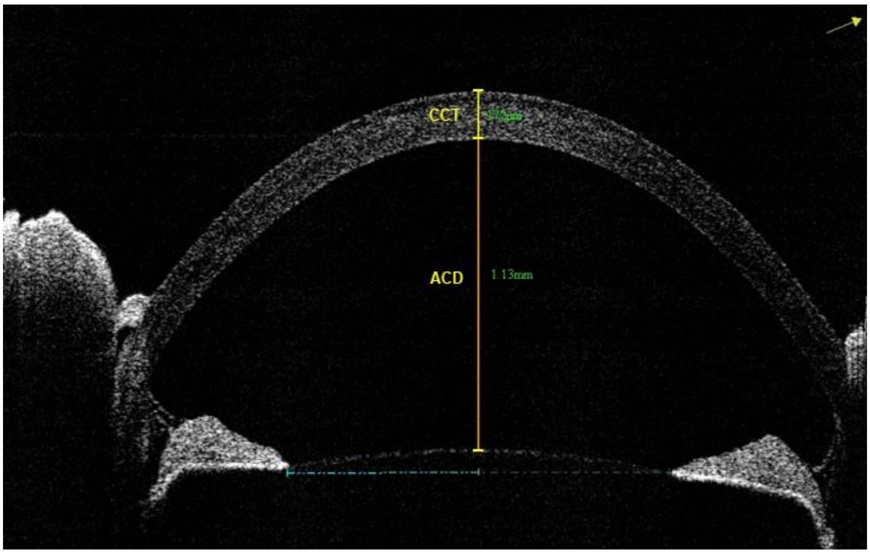

Supplementary Figure S8. A representative example of an anterior segment OCT scan including the measurements of the anterior chamber depth (ACD) and central corneal thickness (CCT) in the control eye of a chicken exposed to SW light (D7).
